# Balancing in- and out-breeding by the predatory mite *Phytoseiulus persimilis*

**DOI:** 10.1007/s10493-018-0225-3

**Published:** 2018-02-19

**Authors:** Demet Atalay, Peter Schausberger

**Affiliations:** 1 Group of Arthropod Ecology and Behavior, Department of Crop Sciences, University of Natural Resources and Life Sciences, Gregor Mendelstrasse 33, 1180 Vienna, Austria; 2 Department of Behavioural Biology, University of Vienna, Althanstrasse 14, 1090 Vienna, Austria

**Keywords:** *Phytoseiulus persimilis*, Predatory mites, Inbreeding depression, Haplo-diploidy, Outbreeding depression

## Abstract

In- and out-breeding depressions are commonly observed phenomena in sexually reproducing organisms with a patchy distribution pattern, and spatial segmentation and/or isolation of groups. At the genetic level, inbreeding depression is due to increased homozygosity, whereas outbreeding depression is due to inferior genetic compatibility of mates. Optimal outbreeding theory suggests that intermediate levels of mate relatedness should provide for the highest fitness gains. Here, we assessed the fitness consequences of genetic relatedness between mates in plant-inhabiting predatory mites *Phytoseiulus persimilis*, which are obligatory sexually reproducing but haplo-diploid. Both females and males arise from fertilized eggs but males lose the paternal chromosome set during embryogenesis, dubbed pseudo-arrhenotoky. *Phytoseiulus persimilis* are highly efficacious in reducing crop-damaging spider mite populations and widely used in biological control. Using iso-female lines of two populations, from Sicily and Greece, we assessed the fecundity of females, and sex ratio of their offspring, that mated with either a sibling, a male from the same population or a male from the other population. Additionally, we recorded mating latency and duration. Females mating with a male from the same population produced more eggs, with a lower female bias, over a longer time than females mating with a sibling or with a male from the other population. Mating latency was unaffected by mate relatedness; mating duration was disproportionally long in sibling couples, likely indicating female reluctance to mate and sub-optimal spermatophore transfer. Our study provides a rare example of in- and out-breeding depression in a haplo-diploid arthropod, supporting the optimal outbreeding theory.

## Introduction

Inbreeding, the mating or breeding between genetically closely related individuals is common in small, fragmented or isolated populations (for review Charlesworth and Charlesworth 1987, 1999; Frankham et al. 2002; Henter 2003). Inbreeding commonly causes reduction in fitness-related measures such as survival, development, growth rates, fecundity, intraspecific competitive ability, and/or tolerance of environmental stressors (Wright 1977; Henter 2003; Radwan 2003; Fox and Reed 2011). Inbreeding can also lead to sperm deformities, sterility and decreased courtship frequencies (Pusey and Wolf 1996). At the genetic level, inbreeding depression is associated with increased homozygosity. Two major hypotheses of the causal mechanisms of inbreeding depression have been put forward, i.e. the partial dominance hypothesis and the over-dominance hypothesis (Charlesworth and Charlesworth 1999; Wright et al. 2008; Charlesworth and Willis 2009). Both hypotheses assume that increased homozygosity reduces fitness. The partial dominance hypothesis states that fitness reduction arising from inbreeding depends on the genetic load of recessive deleterious alleles. Inbreeding increases the expression of deleterious recessive alleles by increasing the frequency of deleterious homozygous combinations (Waller 1993; Shultz and Willis 1995). The overdominance hypothesis states that heterozygotes generally have a superior fitness than either homozygote (Charlesworth and Willis 2009).

Assuming overdominance as causal mechanism, inbreeding depression should not differ between diplo-diploid and haplo-diploid organisms. Assuming partial dominance, inbreeding depression should primarily occur in diplo-diploids rather than haplo-diploids. In haplo-diploids, in which fertilized eggs commonly become diploid females and unfertilized eggs become haploid males, deleterious recessive alleles should be expressed and purged in haploid males (Lande and Schemske 1985; Charlesworth and Charlesworth 1987). In haplo-diploid species, recessive deleterious alleles are exposed to continuous selection in hemizygous (haploid) males, which are thus expected to carry a lower genetic load (Charlesworth et al. 1990; Wang et al. 1999). Nonetheless, albeit at large and averaged across organisms, inbreeding depression is less severe in haplo-diploids than in diplo-diploids (Antolin 1999; Henter 2003), quite a few studies have shown that excessive inbreeding may also cause significant negative effects in haplo-diploids. For example, in parasitoid wasps inbreeding may cause 38% shortening of longevity and 32% lower offspring production (Henter 2003). Similarly, Whitehorn et al. (2009) observed that inbred haplo-diploid bumblebees suffered from reduced growth rates, shortened longevity and lower offspring production.

As compared to inbreeding depression, also mating between genetically distant individuals may be disadvantageous for a variety of reasons, which is accordingly dubbed outbreeding depression (Bateson 1983; Templeton 1986; Pusey and Wolf 1996). Although outbreeding is commonly related to increased fitness relative to excessive inbreeding, it very much depends on the level of the mates’ genetic dissimilarity. Above a critical threshold of genetic dissimilarity of outbreeding mates - in the most extreme case from different species - fitness may be lower than at intermediate levels of genetic dissimilarity (Mitton 1993). Compared to inbreeding depression, experimental documentation of outbreeding depression is scant (e.g. Thornhill 1993; Waser et al. 2000; Peer and Taborsky 2005). The underlying mechanisms of outbreeding depression include underdominance (also called negative overdominance) and deleterious epistatic interactions, resulting in break-down of individual co-adapted gene complexes or, in individuals adapted to local conditions, maladaptation (Charlesworth and Charlesworth 1987; Edmands 2002). Besides genetic costs, outbreeding animals may incur somatic costs, for example, via the risk associated with migration. Migration is usually necessary for the occurrence of outbreeding between individuals of spatially segregated populations of the same species. Migration bears the risk of infection with diseases, lack of familiarity with a local habitat may reduce foraging efficiency, and locally resident conspecifics may attack strangers more severely than familiar individuals (Shields 1993; Pusey and Wolf 1996).

Overall, given the wide range of possible costs and benefits related to different degrees of in- and out-breeding, sexually reproducing organisms should be selected for an optimal balance between in- and out-breeding and achieve the highest fitness at an intermediate genetic distance between mates (Bateson 1983; Shields 1993). Here, we assessed the effects of genetic similarity between mating partners in haplo-diploid plant-inhabiting predatory mites *Phytoseiulus persimilis* Athias-Henriot (Acari, Phytoseiidae). *Phytoseiulus persimilis* are highly specialized predators of herbivorous spider mites, such as two-spotted spider mites *Tetranychus urticae* Koch, which are worldwide some of the most important crop pests (for review McMurtry and Croft 1997). Spider mites are patchily distributed on their host plants; as a consequence, and also because of mutual attraction (Zhang and Sanderson 1992; Strodl and Schausberger 2012, 2013), also their prime natural enemies, *P. persimilis,* are patchily distributed and live in groups. All phytoseiid species incl. *P. persimilis* are sexually reproducing and haplo-diploid; both females and males arise from fertilized eggs, but males lose the paternal chromosome set during embryogenesis, making them haploid (dubbed pseudo-arrhenotoky; Sabelis and Nagelkerke 1988).

Knowledge of in- and out-breeding is an important issue in *P. persimilis* for its unique sex determination system, its relevance as model species in various branches of science and its importance in pest management. However, despite the obvious relevance for the use of predatory mites in basic and applied research, and practical use in biocontrol, inbreeding has rarely been looked at in predatory mites of the family Phytoseiidae. The only report for *P. persimilis* comes from Poe and Enns (1970), who observed that inbred laboratory colonies of *Neoseiulus fallacis* and *P. persimilis* exhibited high mortality in all life stages and lower viability and reduced fecundity in adult females. In contrast, in the predatory mite *Galendromus occidentalis,* nine generations of sibling mating resulted only in mild inbreeding depression (Hoy 1977). In detail, we investigated the mating behavior of *P. persimilis* at different genetic relatedness levels of mates, the resulting fecundity of females, and the sex ratio of their offspring. Our study aimed at scrutinizing the effects of inbreeding versus outbreeding in a haplo-diploid arthropod and thereby contributing to improve the use of *P. persimilis* in both research and biological control.

## Materials and methods

### Experimental animals, population origins and rearing

In the experiments, we used individuals of two different laboratory-reared populations of *P. persimilis*. The populations were founded by specimens field-collected in Sicily and Greece, where *P. persimilis* is native (De Moraes et al. 2004), and maintained in the laboratory for about 6–7 years before starting the experiments (Walzer and Schausberger 2011). The laboratory populations had been founded by 50–100 individuals, and population sizes fluctuated between ca. 50 and a few hundred individuals. In the laboratory, the predators were reared on separate artificial arenas consisting of plastic tiles (15 × 15 × 0.2 cm) resting on water-saturated foam cuboids in plastic boxes (20 × 20 × 6 cm) and surrounded by water-saturated tissue paper. The predators were fed by adding leaves of common bean, *Phaseolus vulgaris* L., infested by *T. urticae* onto the arena at 2- to 3-day intervals (McMurtry and Scriven 1965; Schausberger et al. 2016). *Tetranychus urticae* were reared on whole bean plants *P. vulgaris.* Plants were grown at room temperature, 23 ± 2 °C, and artificial light at 16:8 h L:D photoperiod. All rearing and experimental units were kept in a climate chamber at 25 ± 1 °C, 65 ± 5% RH and 16:8 h L:D photoperiod.

### Pre-experimental procedures

To obtain, rear and test experimental animals, we used detached bean leaf arenas. Each arena consisted of a detached trifoliate bean leaflet placed upside down on a water-saturated foam cuboid kept in a small plastic box (10 × 10 × 6 cm) half-filled with water. Water-saturated tissue paper covered the edges of the leaf to prevent the mites from escaping.

To obtain even-aged eggs of *P. persimilis,* giving rise to iso-female lines (hereafter called families), gravid females were randomly withdrawn from the rearing units and placed on a detached bean leaf arena infested with *T. urticae*. Predator eggs were collected daily, using a fine marten’s hair brush, transferred to new separate leaf arenas infested with *T. urticae*, and reared there until reaching the deutonymphal stage. Deutonymphal females were singly isolated in cylindrical cages of 15 mm diameter and 3 mm height, laser-cut into acrylic plates (75 × 25 mm), closed with fine gauze at the bottom and a microscope slide on the upper side (Schausberger 1997). After reaching adulthood inside the cages, each female was allowed to mate with a male randomly taken from the stock population. Offspring of these couples represented a family and were the individuals used in the experiment. From the egg stage, the offspring of each female (i.e. a family) were split in two groups and reared on two separate arenas, to allow obtaining unfamiliar sibling mates. Two families (iso-female lines) from each population (Sicily and Greece) were used in the experiment.

### Experimental procedure

**Fig. 1:**
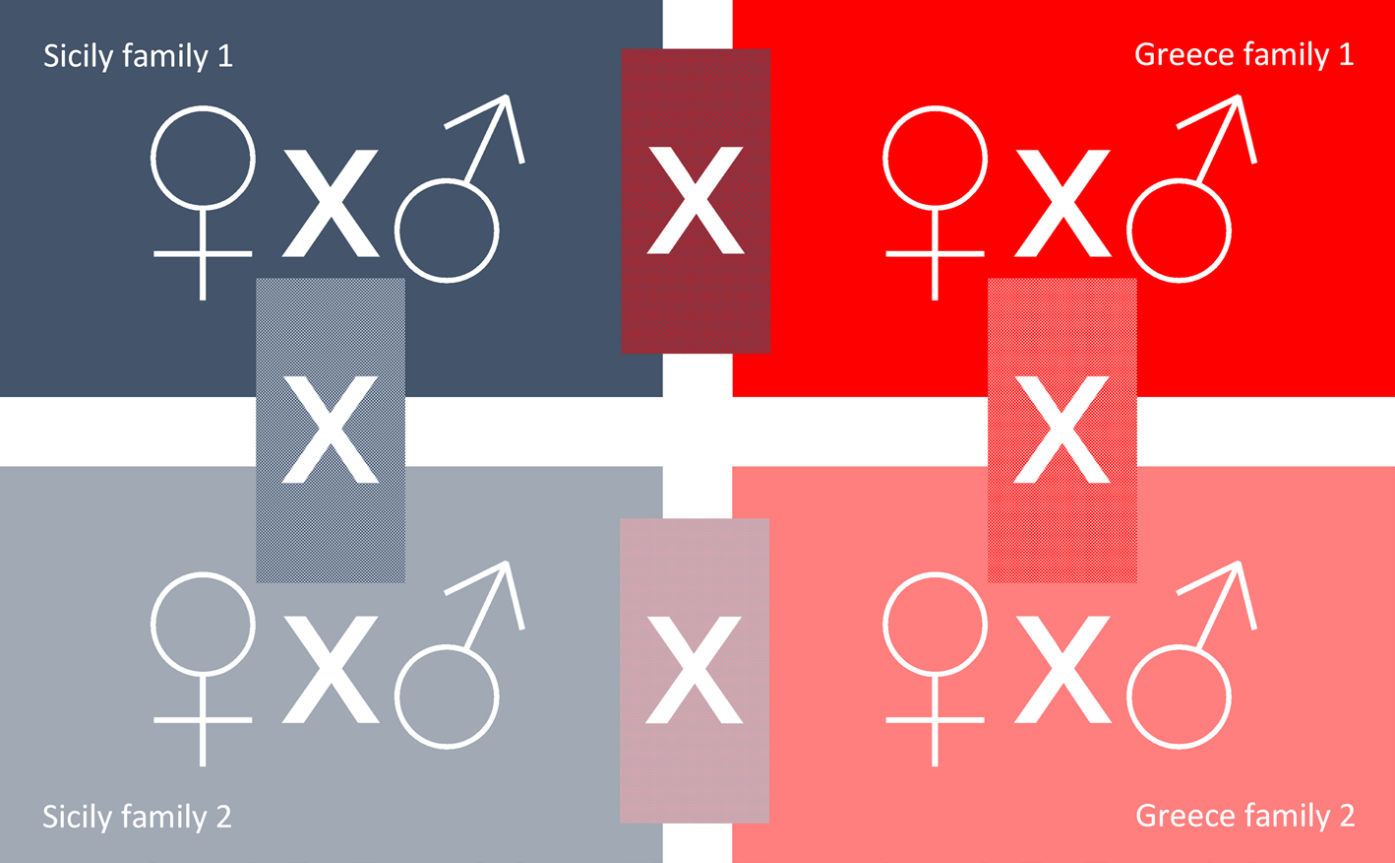
Crossing design used to assess in- and out-breeding depression of *Phytoseiulus persimilis*. Families were iso-female lines established from populations originating from Sicily and Greece. Mates came either from the same family (sibling mating; S), or from different families of the same population (within population; WP), or from different families from different populations (between population; BP)

To assess in- and outbreeding depression, we used three levels of genetic relatedness of mates (close, intermediate and distant), i.e. unfamiliar siblings (S), mates from the same population (within population; WP) and mates from different populations (between population; BP). We paired virgin females from a given family with either a male from the same family (S), or a male from another family from the same population (WP), or a male from a family from the other population (BP) (Fig. 1).

The experiment was started by placing one virgin female and one male, randomly taken from its family, into an acrylic cage, which had been previously loaded with surplus spider mites as prey. Subsequently, we checked the cages every 20 min to record the latency until mating occurred and the mating duration for a total of 4–5 h after placing the couples into the cages. All couples mated successfully and separated again within this time period. Phytoseiid mites mate in the venter-to-venter position, with the male clinging to the female from underneath and transferring the spermatophores with its chelicerae (Amano and Chant 1978). The couples stayed together in their cages for 24 h, with the cages stored upside down on a grid in plastic boxes, the bottom of which was covered by water to elevate the humidity inside the cages (Wimmer et al. 2008). After 24 h, the gravid females were singly transferred onto detached leaf arenas, provided with ample spider mites and left on the arenas until they died of natural causes. Every day we recorded their survival, counted the number of laid eggs, and transferred the eggs onto separate leaf arenas harboring ample spider mites. Eggs collected over a 5-day period were transferred onto the same arena and allowed to grow to adulthood, to determine their sex. Each treatment (S, WP, and BP) was replicated 25–30×.

### Statistical analyses

Statistical analysis was carried out using IBM SPSS 23 (IBM; Armonk, NY, USA). Females not producing a single egg were excluded from analyses, assuming copulation and/or fertilization failure. Such females were rare and equally distributed among the three levels of genetic relatedness (2–4 females per treatment). We compared the time elapsed until mating occurred (mating latency), the duration of mating, the total number of eggs produced by a female and the oviposition period among treatments (level of genetic relatedness of mates) and between populations (Sicily and Greece) by separate generalized linear models (GLM; normal distribution, identity link function). We used full models with two independent main variables, level of mate relatedness and population origin, and their interaction. Least significant difference (LSD) tests were used for post hoc pairwise comparisons between levels of genetic relatedness. The offspring sex ratio was compared among treatments and between populations by GLM (binomial distribution, log-link function).

## Results

**Fig. 2:**
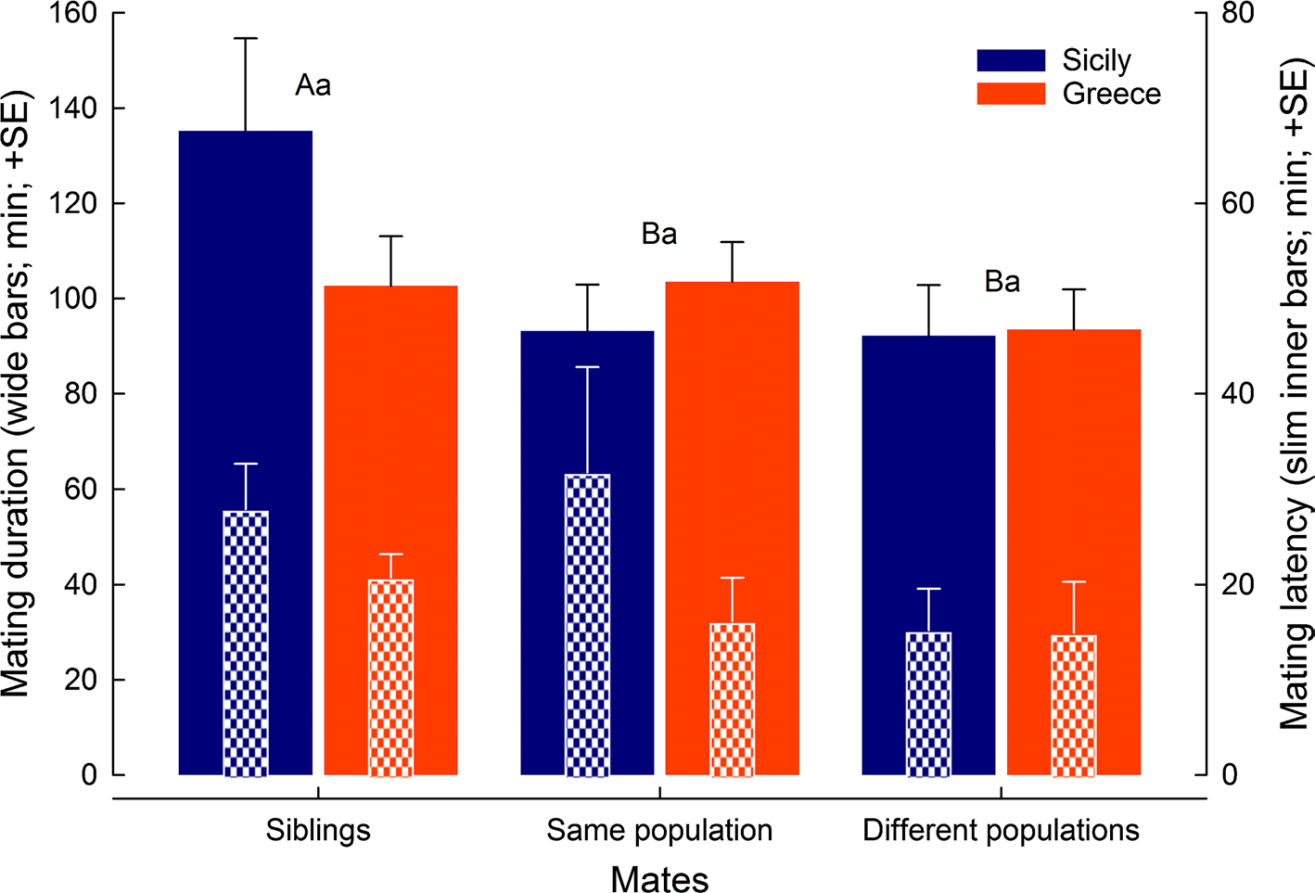
Mating duration and mating latency of *Phytoseiulus persimilis*. Mating partners came either from the same family (siblings), or from different families from the same population (Sicily or Greece), or from different families from different populations (Sicily and Greece). Wide bars represent mating duration, slim inner bars mating latency. Different capital and lower case letters above bars indicate significant differences in mating duration and latency among mate relatedness treatments (LSDs following GLM; *P* < 0.05)

Neither level of genetic relatedness (GLM; Wald χ^2^ = 3.037, *df* = 2, *P* = 0.21) nor population (Wald χ^2^ = 2.475, *df* = 1, *P* = 0.11) nor the interaction between level of relatedness and population (Wald χ^2^ = 1.679, *df* = 2, *P* = 0.43) had an effect on the mating latency (Fig. 2). The level of genetic relatedness affected the mating duration (GLM; Wald χ^2^ = 6.305, *df* = 2, *P* = 0.04), which was unaffected by population (Wald χ^2^ = 0.623, *df* = 2, *P* = 0.73) and the interaction between the level of genetic relatedness and population (Wald χ^2^ = 4.358, *df* = 2, *P* = 0.11) (Fig. 2): sibling (S) couples mated longer than WP and BP couples (LSD; *P* ≤ 0.05), while WP and BP couples mated similarly long (*P* > 0.05).

**Fig. 3:**
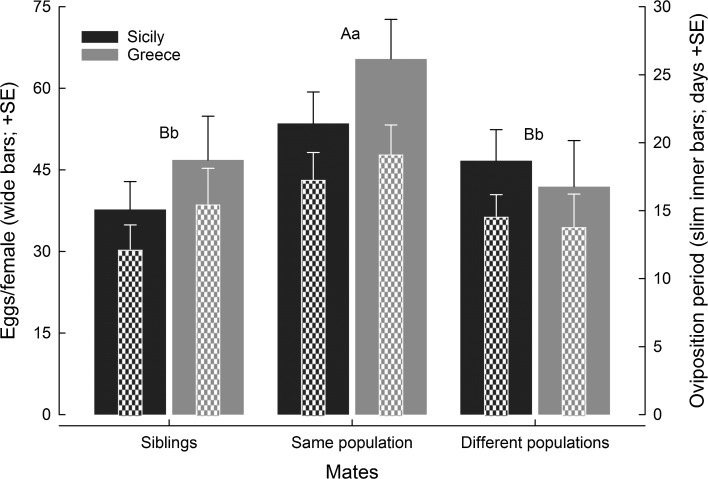
Total number of eggs and oviposition period of *Phytoseiulus persimilis*. Mating partners came either from the same family (siblings), or from different families from the same population (Sicily or Greece), or from different families from different populations (Sicily and Greece). Wide bars represent the number of eggs, slim inner bars represent the oviposition period. Different capital and lower case letters above bars indicate significant differences in number of eggs and oviposition period among mate relatedness treatments (LSDs following GLM; *P* < 0.05)

The level of genetic relatedness affected fecundity (GLM; Wald χ^2^ = 7.141, *df* = 2, *P* = 0.02) and the oviposition period (Wald χ^2^ = 5.674, *df* = 2, *P* = 0.05), irrespective of population (Wald χ^2^ = 0.713, *df* = 2, *P* = 0.39 and Wald χ^2^ = 0.744, *df* = 2, *P* = 0.38) and the interaction between level of genetic relatedness and population (Wald χ^2^ = 0.700, *df* = 2, *P* = 0.42 and Wald χ^2^ = 0.936, *df* = 2, *P* = 0.62) (Fig. 3). In both populations, WP females produced in total more eggs over a longer time than BP and S females (LSD; *P* ≤ 0.05 for each pairwise comparison); BP and S females produced similar numbers of eggs and had similarly long oviposition periods (*P* > 0.05) (Fig. 3).

Offspring sex ratio was influenced by both the level of genetic relatedness (GLM; Wald χ^2^ = 17.816, *df* = 2, *P* < 0.001) and population (Wald χ^2^ = 6.515, *df* = 2, *P* = 0.01) as main effects, but not the interaction of level of genetic relatedness and population (Wald χ^2^ = 0.345, *df* = 2, *P* = 0.84). Offspring of females from Greece (mean female proportion ± SE; 0.82 ± 0.01) were more female-biased than offspring of females from Sicily (0.78 ± 0.01). Offspring of WP females (0.76 ± 0.01) were less female-biased than offspring of S (0.81 ± 0.01) and BP (0.83 ± 0.01) females.

## Discussion

We assessed excessive in- and outbreeding in *P. persimilis* by comparing the behavioral and reproductive performance of couples having one of three levels of genetic relatedness, that is, mating between siblings, between individuals of the same population and between individuals of different populations. Our experiment revealed a fitness decrease in both closely inbreeding and distantly outbreeding females, suggesting an optimal balance of genetic relatedness of mates belonging to the same population. Sib-mating females and females mating with a male from a different population produced fewer eggs over a shorter time period than females mating with a male from the same population. Mating duration was longer, and the proportion of daughters higher, in sib-mating than within and between population mating couples.

Inbreeding depression is often determined by measuring reproduction after a single generation of inbreeding (Charlesworth and Willis 2009), with many such studies showing analogous results to our study, i.e. inbreeding reducing fecundity (Pusey and Wolf 1996; Saito et al. 2000; Keller and Waller 2002; Swindell and Bouzat 2006; Charlesworth et al. 2007). For pseudo-arrhenotokous predatory mites, Poe and Enns (1970) reported that inbreeding by *P. persimilis* and another predatory mite, *N. fallacis,* resulted in strongly reduced egg production and decreased hatching success of offspring. In contrast, Hoy (1977) observed only mild inbreeding depression in the predatory mite *G. occidentalis*; females from inbred lines had slightly shorter longevity and lower egg production compared to females from outbred lines. In diplo-diploid bulb mites *Rhizoglyphus robini*, Radwan (2003) observed that sib-mating severely reduced fecundity in F_1_ offspring. In truly arrhenotokous spider mites *T. urticae*, Vala et al. (2003) observed that sib-mating increased mortality, shortened longevity and strongly reduced fecundity in F_1_ offspring. Similarly, Perrot-Minnot et al. (2004) observed lower fecundity and higher offspring mortality in inbred than outbred *T. urticae*.

Mating latency did not depend on the level of relatedness. However, this may have been due to the no-choice situation and the small size and homogeneity of the experimental cages, promoting mate encounter and generally shortening the latencies, as compared to leaves. On leaf arenas, which are more heterogeneous and where mate finding is more difficult, mating latency has been shown being an appropriate indicator of mate choice. For example, Enigl and Schausberger (2004) observed that virgin *P. persimilis* females tended to accept unrelated mates earlier than related mates. The mean mating duration exceeded in all three treatments (level of genetic relatedness of couples) of both populations the species-specific threshold needed for complete successful insemination, which is about 80 min at 25 °C (Walzer and Schausberger 2015). Below this threshold, the mating duration is positively linked to the volume of the endo-spermatophores following spermathecal insemination (Amano and Chant 1978; Schulten 1985). The significantly longer mating duration in sib-mating couples than in within- and between-population couples thus likely reflects decreased behavioral compatibility of sib couples, for example, because of female reluctance to allow unrestricted filling of both spermathecae, and thus suboptimal spermatophore transfer by the males (Schausberger et al. 2017).

The offspring sex ratio of predatory mites is mostly female-biased, usually at around two females to one male, under favorable conditions (Hoy 1977; Dyer and Swift 1979; Walter 1992). In many animal taxa, the offspring sex ratio is influenced by the level of inbreeding, with a positive correlation between the level of inbreeding and the male bias among offspring (Greeff and Taylor 1997; Frankham and Wilcken 2006). However, in view of kin selection theory, to reduce local mate competition among sons (Hamilton 1967), females mating with a close relative should reduce the male bias and close inbreeding should hence result in more female-biased offspring sex ratios (West et al. 2002). In haplo-diploid species, only daughters will be affected by homozygosity problems arising from inbreeding while sons will be unaffected (Antolin 1999; Henter 2003). Hoy (1977) reported that *G. occidentalis* showed only mild inbreeding depression after nine generations without any alterations of offspring sex ratio. In contrast, in our study both sib-mating females and between-population mating females shifted the offspring sex ratio towards daughters. Reducing local mate competition as ultimate explanation is only applicable to sib-mating females; an alternative or additional explanation, applying to both sib-mating and between-population mating females, is mothers counterbalancing the fitness decrease of individual daughters, accruing from reduced fecundity, by increasing the proportion of daughters among offspring.

In summary, our findings support the hypothesis of optimal outbreeding (Bateson 1983). Both excessive inbreeding and distant outbreeding reduced the fitness of *P. persimilis,* relative to an intermediate level of mate relatedness. Nonetheless, our study only quantified the effects of mate relatedness in the F_1_ generation. To gain a more comprehensive understanding, follow-up studies should scrutinize long-term trans-generational consequences of founder relatedness effects and chronic inbreeding on population growth and persistence. Taken together, our outcomes should foster the use of *P. persimilis* as model species in studies on in- and out-breeding by haplo-diploid organisms (Henter 2003). The outcomes should contribute to improve the use of *P. persimilis* in both fundamental and applied research, for example, by optimizing laboratory and commercial rearing, and allow tailoring efficient release and establishment strategies in augmentative and classical biocontrol.

## References

[CR1] Amano H, Chant DA (1978). Mating behaviour and reproductive mechanisms of two species of predacious mites, *Phytoseiulus persimilis* Athias-Henriot and *Amblyseius andersoni* (Chant) (Acarina: Phytoseiidae). Acarologia.

[CR2] Antolin MF (1999). A genetic perspective on mating systems and sex ratios of parasitoid wasps. Res Popul Ecol.

[CR3] Bateson P (1983). Optimal outbreeding.

[CR4] Charlesworth D, Charlesworth B (1987). Inbreeding depression and its evolutionary consequences. Annu Rev Ecol Syst.

[CR5] Charlesworth B, Charlesworth D (1999). The genetic basis of inbreeding depression. Genet Res Camb.

[CR6] Charlesworth D, Willis JH (2009). The genetics of inbreeding depression. Nat Rev Genet.

[CR7] Charlesworth D, Morgan MT, Charlesworth B (1990). Inbreeding depression, genetic load and the evolution of outcrossing rates in a multi-locus system with no linkage. Evolution.

[CR8] Charlesworth B, Miyo T, Borthwick H (2007). Selection responses of means and inbreeding depression for female fecundity in *Drosophila melanogaster* suggest contributions from intermediate-frequency alleles to quantitative trait variation. Genet Res.

[CR9] De Moraes GJ, McMurtry JA, Denmark HA, Campos CB (2004). A revised catalogue of the mite family Phytoseiidae. Zootaxa.

[CR10] Dyer J, Swift FC (1979). Sex ratio in field populations of phytoseiid mites (Acarina: Phytoseiidae). Ann Entomol Soc Am.

[CR11] Edmands S (2002). Does parental divergence predict reproductive compatibility?. Trends Ecol Evol.

[CR12] Enigl M, Schausberger P (2004). Mate choice in the predaceous mite *Phytoseiulus persimilis*: evidence of self-referent phenotype matching?. Entomol Exp Appl.

[CR13] Fox CW, Reed DH (2011). Inbreeding depression increases with environmental stress: an experimental study and meta-analysis. Evolution.

[CR14] Frankham R, Wilcken J (2006). Does inbreeding distort sex-ratios?. Conserv Genet.

[CR15] Frankham R, Briscoe DA, Ballou JD (2002). Introduction to conservation genetics.

[CR16] Greeff JM, Taylor PD (1997). Effects of inbreeding depression on relatedness and optimal sex ratios. Evol Ecol.

[CR17] Hamilton WD (1967). Extraordinary sex ratios. Science.

[CR18] Henter HJ (2003). Inbreeding depression and haplodiploidy: experimental measures in a parasitoid and comparisons across diploid and haplodiploid insect taxa. Evolution.

[CR19] Hoy MA (1977). Inbreeding in the arrhenotokous predator *Metaseiulus occidentalis* (Nesbitt) (Acari: Phytoseiidae). Int J Acarol.

[CR20] Keller LF, Waller DM (2002). Inbreeding effects in wild populations. Trends Ecol Evol.

[CR21] Lande R, Schemske DW (1985). The evolution of self-fertilization and inbreeding depression in plants. I. Genetic models. Evolution.

[CR22] McMurtry JA, Croft BA (1997). Life-styles of phytoseiid mites and their roles in biological control. Annu Rev Entomol.

[CR23] McMurtry JA, Scriven GT (1965). Insectary production of phytoseiid mites. J Econ Entomol.

[CR24] Mitton JB, Thornhill NW (1993). Theory and data pertinent to the relationship between heterozygosity and fitness. The natural history of inbreeding and outbreeding.

[CR25] Peer K, Taborsky M (2005). Outbreeding depression, but no inbreeding depression in haplodiploid ambrosia beetles with regular sibling mating. Evolution.

[CR26] Perrot-Minnot MJ, Migeon A, Navajas M (2004). Intergenomic interactions affect female reproduction: evidence from introgression and inbreeding depression in a haplodiploid mite. Heredity.

[CR27] Poe SL, Enns WR (1970). Effects of inbreeding on closed populations of predaceous mites (Acarina: Phytoseiidae). Can Entomol.

[CR28] Pusey A, Wolf M (1996). Inbreeding avoidance in animals. Trends Ecol Evol.

[CR29] Radwan J (2003). Inbreeding depression in fecundity and inbred line extinction in the bulb mite, *Rhizoglyphus robini*. Heredity.

[CR30] Sabelis MW, Nagelkerke CJ (1988). Evolution of pseudo-arrhenotoky. Exp Appl Acarol.

[CR31] Saito Y, Sahara K, Mori K (2000). Inbreeding depression by recessive deleterious genes affecting female fecundity of a haplo-diploid mite. J Evol Biol.

[CR32] Schausberger P (1997). Inter-and intraspecific predation on immatures by adult females in *Euseius finlandicus*, *Typhlodromus pyri* and *Kampimodromus aberrans* (Acari: Phytoseiidae). Exp Appl Acarol.

[CR33] Schausberger P, Patiño-Ruiz JD, Osakabe M, Murata Y, Sugimoto N, Uesugi R, Walzer A (2016). Ultimate drivers and proximate correlates of polyandry in predatory mites. PLoS ONE.

[CR34] Schausberger P, Walzer A, Murata Y, Osakabe M (2017). Low level of polyandry constrains phenotypic plasticity of male body size in mites. PLoS ONE.

[CR35] Schulten GGM, Helle W, Sabelis MW (1985). Mating. Spider mites, their biology, natural enemies and control.

[CR36] Shields WM, Thornhill NW (1993). The natural and unnatural history of inbreeding and outbreeding. The natural history of inbreeding and outbreeding: theoretical and empirical perspectives on population structure.

[CR37] Shultz S, Willis JH (1995). Individual variation in inbreeding depression: the roles of inbreeding history and mutation. Genetics.

[CR38] Strodl MA, Schausberger P (2012). Social familiarity modulates group living and foraging behaviour of juvenile predatory mites. Naturwissenschaften.

[CR39] Strodl MA, Schausberger P (2013). Social familiarity relaxes the constraints of limited attention and enhances reproduction of group-living predatory mites. Oikos.

[CR40] Swindell WR, Bouzat JL (2006). Selection and inbreeding depression: effects of inbreeding rate and inbreeding environment. Evolution.

[CR41] Templeton AR, Soulé ME (1986). Coadaptation and outbreeding depression. Conservation biology: the science of scarcity and diversity.

[CR42] Thornhill NW (1993). The natural history of inbreeding and outbreeding: theoretical and empirical perspectives.

[CR43] Vala F, Breeuwer JAJ, Sabelis MW (2003). Sorting out the effects of *Wolbachia*, genotype and inbreeding on life-history traits of a spider mite. Exp Appl Acarol.

[CR44] Waller DM, Thornhill NW (1993). The statics and dynamics of mating system evolution. The natural history of inbreeding and outbreeding.

[CR45] Walter DE (1992). Leaf surface structure and the distribution of *Phytoseius* mites (Acarina: Phytoseiidae) in south-eastern Australian forests. Aust J Zool.

[CR46] Walzer A, Schausberger P (2011). Sex-specific developmental plasticity of generalist and specialist predatory mites (Acari: Phytoseiidae) in response to food stress. Biol J Linn Soc.

[CR47] Walzer A, Schausberger P (2015). Interdependent effects of male and female body size plasticity on mating behaviour of predatory mites. Anim Behav.

[CR48] Wang J, Hill WG, Charlesworth D, Charlesworth B (1999). Dynamics of inbreeding depression due to deleterious mutations in small populations: mutation parameters and inbreeding rate. Genet Res.

[CR49] Waser NM, Price MV, Shaw RG (2000). Outbreeding depression varies among cohorts of *Ipomopsis aggregata* planted in nature. Evolution.

[CR50] West SA, Reece SE, Sheldon BC (2002). Sex ratios. Heredity.

[CR51] Whitehorn PR, Tinsley MC, Brown MJ, Darvil B, Goulson D (2009). Impacts of inbreeding on bumblebee colony fitness under field conditions. BMC Evol Biol.

[CR52] Wimmer D, Hoffmann D, Schausberger P (2008). Prey suitability of western flower thrips, *Frankliniella occidentalis*, and onion thrips, *Thrips tabaci*, for the predatory mite *Amblyseius swirskii*. Biocont Sci Techn.

[CR53] Wright S (1977). Evolution and the genetics of populations, vol 3: experimental results and evolutionary deductions.

[CR54] Wright LI, Tregenza T, Hosken DJ (2008). Inbreeding, inbreeding depression and extinction. Conserv Genet.

[CR55] Zhang Z-Q, Sanderson JP (1992). Short distance location of spider mite colonies by three predatory mites (Acari, Tetranychidae, Phytoseiidae)—predator responses to prey-associated and predator-associated stimuli. Environ Entomol.

